# Supplementary material for: 4E Interacting Protein as a Potential Novel Drug Target for Nucleoside Analogues in Trypanosoma brucei
Source: Microorganisms. 2021 Apr 13;9(4):826. doi: 10.3390/microorganisms9040826 (PMC8069773; doi:10.3390/microorganisms9040826)
Supplement: Supplementary file 1 [file microorganisms-09-00826-s001.pdf]

Supplementary material 1: Synthesis of compound **4**.

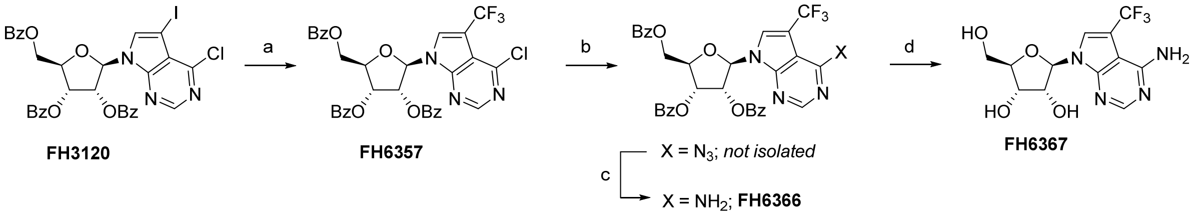

Scheme S1. Reagents and conditions: a) $TMSCF_3$, KF, $B(OMe)_3$, CuI, 1,10-phenanthroline, DMSO, 60 °C; b) NaN_3, DMF, 65 °C; c) 1. 1M PMe_3 in THF, THF; 2. 1M aq. HOAC, MeCN, 65 °C, 20 % (over 3 steps); d) 7N NH_3/MeOH, 87 %;.

General experimental

All reagents and solvents were obtained from standard commercial sources and were of analytical grade. Unless otherwise specified, they were used as received. All moisture sensitive reactions were carried out under argon atmosphere. Reactions were carried out at ambient temperature, unless otherwise indicated. Analytical TLC was performed on Machery-Nagel® precoated F254 aluminum plates and were visualized by UV followed by staining with basic aq. $KMnO_4$, Cerium-Molybdate, or sulfuric acid-anisaldehyde spray. Column chromatography was performed using a Reveleris X2 (Grace/Büchi) automated Flash unit employing pre-packed silica columns. Exact mass measurements were performed on a Waters LCT Premier XE™ Time of Flight (ToF) mass spectrometer equipped with a standard electrospray (ESI) and modular Lockspray™ interface. Samples were infused in a MeCN / water (1:1) + 0.1 % formic acid mixture at 100 µL / min. NMR spectra were recorded on a Varian Mercury 300 MHz spectrometer. Chemical shifts (δ) are given in ppm and spectra are referenced to the residual solvent peak. Coupling constants are given in Hz. In ^{19}F-NMR, signals were referenced to $CDCl_3$ or DMSO-d_6 lock resonance frequency according to IUPAC referencing with $CFCl_3$ set to 0 ppm. Melting points were determined on a Büchi-545

apparatus and are uncorrected. Purity was assessed by means of analytical LC-MS employing a Waters Alliance 2695 XE separation Module using a Phenomenex Luna® reversed-phase C18 (2) column (3 µm, 100x2.00 mm) and a gradient system of HCOOH in H2O (0.1 %, v/v)/HCOOH in MeCN (0.1 %, v/v) at a flow rate of 0.4 mL / min, 10:90 to 0:100 in 9 minutes. High-resolution MS spectra were recorded on a Waters LCT Premier XE Mass spectrometer. The obtained final compound had a purity of >95%, as assayed by analytical HPLC (UV detection).

Compound **FH3120** was prepared according to the following reference:

Seela, F., Ming, X., Tetrahedron 2007, 63 (39), 9850-9861, doi: 10.1016/j.tet.2007.06.107.

4-Chloro-5-trifluoromethyl-N7-(2',3',5'-tri-*O*-benzoyl-β-D-ribofuranosyl)-pyrrolo[2,3-*d*]pyrimidine (FH6357) In a flame-dried culture tube, equipped with a stir bar, was added under argon: **FH3120** (0.72 g, 1.0 mmol, 1 eq.), CuI (0.038 g, 0.20 mmol, 0.2 eq.), 1,10-phenanthroline (0.036 g, 0.20 mmol, 0.2 eq.) and KF (0.17 g, 3.0 mmol, 3 eq.). Then, the culture tube was capped with a septum and evacuated. Next, the flask was refilled with argon. This procedure was repeated three times in total. Then, anhydrous DMSO (2.0 mL, 2.0 mL / mmol SM) was added, followed by $B(OMe)_3$ (0.33 mL, 3.0 mmol, 3 eq.) and $TMSCF_3$ (0.45 mL, 3.0 mmol, 3 eq.). The mixture was stirred at ambient temperature for 1-2 min to ensure adequate homogenization, and then transferred to a pre-heated oil bath at 60 °C. After 18H, the reaction mixture was cooled to ambient temperature, and water added. Next, EA was added, and the layers were separated. The water layer was extracted twice more with EA. The organic layers were combined, dried over Na_2SO_4, filtered and evaporated till dryness. The residue was purified by column chromatography 0 → 20 % EA / hexanes to give **FH6357** (0.29 g). The product still contained (~10 %) of unreacted iodide SM; and was therefore directly used in the next steps (azidation and Staudinger reduction).

4-Amino-5-trifluoromethyl-N7-(2',3',5'-tri-*O*-benzoyl-β-D-ribofuranosyl)-pyrrolo[2,3-*d*]pyrimidine (FH6366) The mixture containing **FH6357** (0.29 g) was dissolved in anhydrous DMF (10 mL / mmol SM). Next, NaN_3 (2.05 eq.) was added. The resulting mixture was heated in a pre-heated oil bath at 65 °C for 30 min. Next, the mixture was cooled to ambient temperature. Then, the mixture was poured into half-saturated $NaHCO_3$ solution and EA (equal volumes). The layers were separated, and the water layer extracted two more times with EA. The organic layers were combined, dried over Na_2SO_4, filtered and evaporated till dryness. The residue was purified by column chromatography (generally a gradient of 10 → 35 % EA / Hexanes or PET) to yield the protected azido-nucleoside. The **azido-nucleoside** (1 eq.) was dissolved in THF (10 mL / mmol). Then, PMe_3 solution (1M in THF; 2.7 eq.) was added and the mixture stirred at ambient temperature until TLC analysis showed full conversion of starting material (generally 1 h). Next, the solution was evaporated till dryness, and subsequently re-dissolved in MeCN (10 mL / mmol). To this solution was added a 1M aq. HOAc solution (3.3 eq.), and the mixture heated in a pre-heated oil bath at 65 °C for 1H. Next, the mixture was cooled to ambient temperature and poured into sat. aq. $NaHCO_3$ solution. DCM was added, layers were separated, and the water layer extracted two more times with DCM. The organic layers were combined, dried over Na_2SO_4, filtered and evaporated till dryness. Purification by column chromatography (a gradient of 40 → 75 % EA / hexanes). As such **FH6357** (0.289 g) gave rise to **FH6366** (0.132 g, 0.204 mmol) as a white foam in 20 % yield over three steps. ^{1}H NMR (300 MHz, $CDCl_3$) δ: 4.70 (dd, *J* = 12.0, 3.9 Hz, 1H, H-5''), 4.78 – 4.81 (m, 1H, H-4'), 4.89 (dd, *J* = 12.0, 3.0 Hz, 1H, H-5'), 5.52 (br. s, 2H, NH_2), 6.11 – 6.18 (m, 2H, H-3', H-2'), 6.66 (d, *J* = 5.1 Hz, 1H, H-1'), 7.33 – 7.64 (m, 11H, OBz, H-6), 7.93 – 7.96 (m, 2H, OBz), 7.97 – 8.01 (m, 2H, OBz), 8.10 – 8.14 (m, 2H, OBz), 8.33 (s, 1H, H-2). ^{19}F-NMR (282 MHz, $CDCl_3$) δ: -55.7. ^{13}C NMR (75 MHz, $CDCl_3$) δ: 63.9 (C-5'), 71.7 (C-3'), 74.5 (C-2'), 80.9 (C-4'), 87.0 (C-1'), 99.9 (s, 1C, C-4a), 106.4 (q, *J* = 37.8 Hz, 1C, C-5), 123.0 (q, *J* = 5.7 Hz, 1C, C-6),

123.2 (d, J = 264.4 Hz, 1C, CF_3), 128.72, 128.75, 128.79, 128.9, 129.0, 129.5, 129.9, 130.09, 130.14, 133.8, 133.97, 134.0, 152.0 (C-7a), 153.5 (C-2), 156.2 (C-4), 165.4 (C=O), 165.6 (C=O), 166.4 (C=O). HRMS (ESI): calculated for $C_{33}H_{26}F_3N_4O_7$ ($[M+H]^+$): 647.1748, found: 647.1745.

4-Amino-5-trifluoromethyl-N7-(β-D-ribofuranosyl)-pyrrolo[2,3-*d*]pyrimidine (FH6367)

FH6366 (0.12 g, 0.19 mmol) was dissolved in 7N NH_3 / MeOH and stirred at ambient temperature overnight. next, the mixture was evaporated till dryness. The residue was purified by column chromatography 0 → 7.5 % MeOH / DCM to give **FH6367** (0.055 g, 0.17 mmol) as a white solid in 87 % yield. Melting point: 195 °C. ^1H NMR (300 MHz, DMSO-d_6) δ: 3.52 – 3.59 (m, 1H, H-5''), 3.63 – 3.70 (m, 1H, H-5'), 3.92 (dd, J = 6.9, 3.6 Hz, 1H, H-4'), 4.09 (t, J = 5.1 Hz, 1H, H-3'), 4.40 (dd, J = 11.4, 6.0 Hz, 1H, H-2'), 5.14 (d, J = 4.8 Hz, 1H, OH-3'), 5.20 (d, J = 5.4 Hz, 1H, OH-5'), 5.40 (d, J = 6.0 Hz, 1H, OH-2'), 6.10 (d, J = 6.0 Hz, 1H, H-1'), 6.60 (br. s, 2H, NH_2), 8.19 (d, J = 1.5 Hz, 1H, H-6), 8.23 (s, 1H, H-2). ^{19}F-NMR (282 MHz, DMSO-d_6) δ: -53.7. ^{13}C NMR (75 MHz, DMSO-d_6) δ: 61.2 (C-5'), 70.2 (C-3'), 74.0 (C-2'), 85.3 (C-4'), 87.3 (C-1'), 98.0 (C-4a), 103.3 (q, J = 36.7 Hz, 1C, C-5), 123.5 (q, J = 264.5 Hz, 1C, CF_3), 124.2 (q, J = 6.9 Hz, 1C, C-6), 151.3 (C-7a), 153.0 (C-2), 156.2 (C-4). HRMS (ESI): calculated for $C_{12}H_{14}F_3N_4O_4$ ([M+H]+): 335.0962, found: 335.0958.

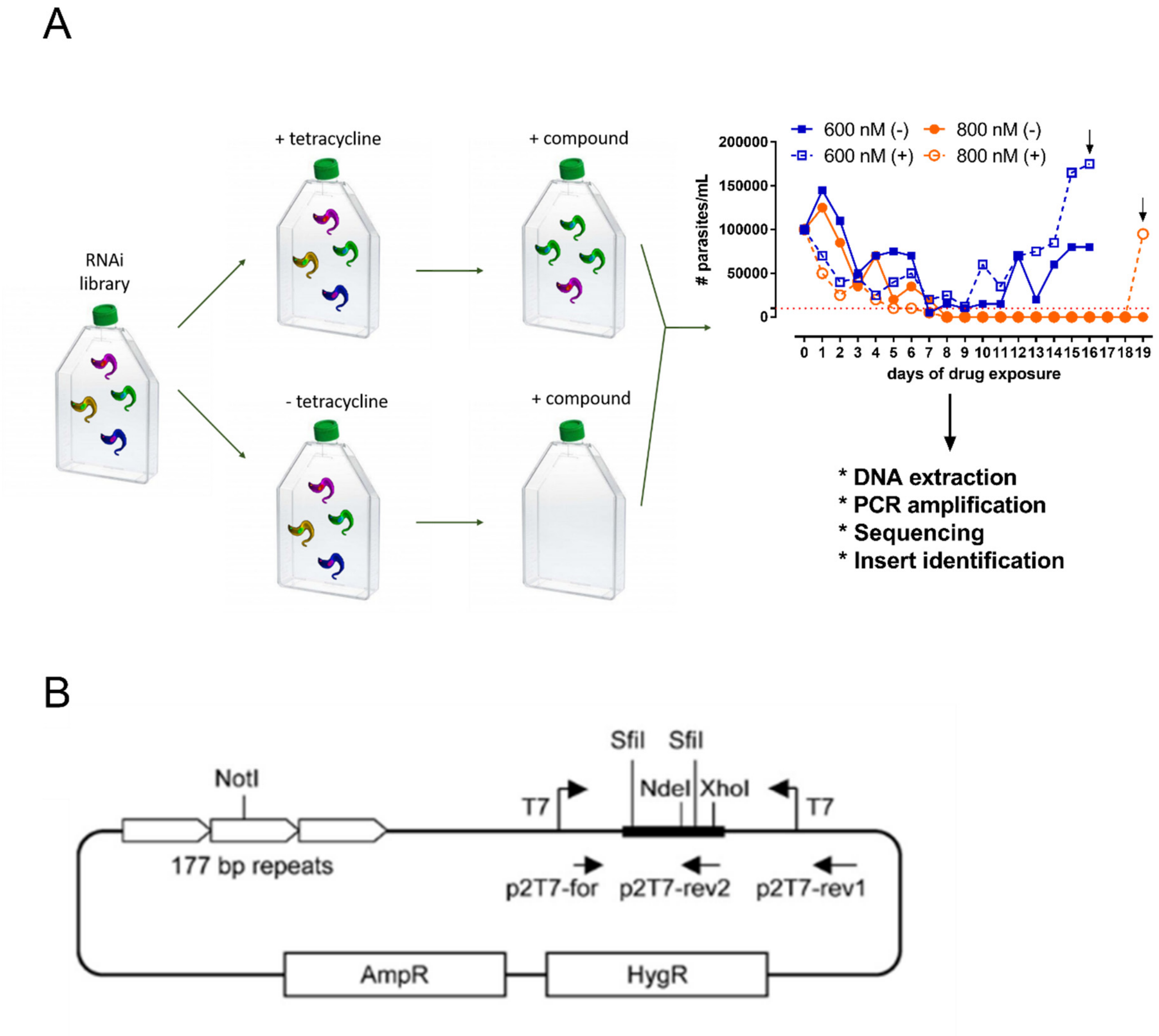

Figure S1. Overview of the RNAi screening protocol. (**A**) Experimental overview of the screening protocol of RNAi libraries for compound **5**. (**B**) Graphic representation of the P2T7Bern vector [25].

Table S1. Overview of the used primers.

Name	Sequence	Tm (°C)[a]
TERT_F	GAGCGTGTGACTTCCGAAGG	58.1
TERT_R	AGGAACTGTCACGGAGTTTGC	57.6
H2B_F	CACCGAACTCTCCGTCAAGT	56.8
H2B_R	AGCCTGAATTTTCCCGTACA	54.2
ActinA_F	GTACCACTGGCATTGTTCTCG	56.0
ActinA_R	CTTCATGAGATATTCCGTCAGGTC	54.8
Eef2_F	TGTCAGTCATCGCCCATGTG	57.6
Eef2_R	CATCCTTGCGAGTGTCAGTGA	57.1
18S_tryp_F	ACGGAATGGCACCACAAGAC	58.1
18S_tryp_R	GTCCGTTGACGGAATCAACC	56.2
p2T7_seq	CCGCTCTAGAACTAGTGGA	52.9
p2T7hygPJ4	GGAAAGCTAGCTTGCATGCCTG	58.9
p2T7linker_rev	AGGGCCAGTGAGGCCTCTAGAG	62.4
ADKIN_F	CGTGAGGTGGATGGACTTTT	55.0
ADKIN_R	TTGCAATCTCCTCGACACAG	54.9
EndoG_F	ACGTACCGCAGGAATGTTTC	55.4
EndoG_R	CACTTCTGCTGCTGTTCTGC	56.7
FLA1BP_F	GGACAGCGGTGTCTTCTCTC	57.5
FLA1BP_R	TCCCACTTCACACGTCCATA	55.7
4EIP_F	CTTCTCTGGGGCAAACTCTG	55.6
4EIP_R	CACGGGTCTTTGACCTGATT	55.0
SL-tr_F	AACTAACGCTATTATTAGAA	43.4
SL-tr_R	CAATATAGTACAGAAACTG	42.1

[a]Melting temperature of primers.

Table S2. Overview of the sequencing results of individual RNA inserts obtained following selection of a genome-wide *T.b.brucei* RNAi library exposed to **5**.

Primer[a]	Gene	Gene product	Annotation	Location RNAi	RNAi match
M13-F	Tb927.8.4050	FLA1-binding protein	Tb927_08_v5.1:1203333-1205585(-)	2279-3019	730/741
M13-R	Tb927.8.4040	endonuclease G, putative	Tb927_08_v5.1:1200841-1202361(-)	1-347	344/347
M13-F	Tb927.6.2300	adenosine kinase, putative	Tb927_06_v5.1:718416-719453(+)	500-1201	690/702
M13-R	Tb927.6.2300	adenosine kinase, putative	Tb927_06_v5.1:718416-719453(+)	500-1201	690/702
M13-F	Tb927.4.5500	variant surface glycoprotein, degenerate	Tb927_04_v5.1:1501217-1502767(-)	810-1100	262-291
M13-R	Tb927.4.5500	variant surface glycoprotein, degenerate	Tb927_04_v5.1:1501217-1502767(-)	810-1100	262-291
M13-F	Tb927.9.11050	4E-interacting protein	Tb927_09_v5.1:1742654-1744291(-)	2132-3269	1079/1144
M13-R	Tb927.9.11050	4E-interacting protein	Tb927_09_v5.1:1742654-1744291(-)	1842-2946	1064/1108

[a]M13-F = forward primer; M13-R = reverse primer.
